# Seasonable Decorations

**Published:** 1886-12-18

**Authors:** 


### For the Children's Ward:

SEASONABLE DECORATIONS.

**Something like a Tree.**

One of the lady nurses at the Boston Cottage Hospital informs me that the four wards of the building are kept supplied all the year round with a nice fresh plant by four good Samaritans in the neighbourhood, who never fail to produce a new flower when the last one gets *passé*. A simple, pleasure-giving method, within most people's powers of accomplishment. Let me recommend it to the readers of The Hospital, more especially as the season draws nigh when all and sundry must feel their charitable impulses accentuated. There is a species of decoration which, at this time, should be found in every ward, not only in hospitals and infirmaries, but in the workhouses too, and might so easily be so if people would promptly set their wits and their fingers to work. The ornament is somewhat large, and is usually to be seen in a big round or square tub, standing well in the middle of the room, or in conspicuous dignity at one end. Many are the peculiarities of this particular plant, known botanically by the name of *Abies nigra* or *alba*. First of all, it is a direct contradiction of the old proverb of "too many cooks," etc., because, although one pair of hands might be found equal to its management, it is all the better for the application of a good many. A very funny thing about it is, that it meets the requirements of everybody, old and young alike (probably the only thing in all the world that does! on account of the difference of individual tastes. I know of a lady who likes the smell of damp and escaped gas, and several who don't!). Again, it closely resembles that remarkable plant whose blossoms are only visible to those who sit up all night to behold them, because at night and by lamplight only does this conifer burst into full splendour. There may be frost in June; and the Glastonbury thorn may flower at Christmas-tide; but *this* consistent specimen is never seen in perfection except in the depth of winter! and when it does greet the human eye, what a sight it is! Glittering with snow and icicles, and yet at the same time such a glare of light that a conflagration seems almost unavoidable; and what is odder still, and quite out of keeping with Nature's usual routine, apples and oranges hang from its branches together. Not proving exactly that apples as a rule grow from orange-pips, but just showing that Santa Claus has a mysterious way of doing things, and his especial tree produces everything conceivable at the same time, while animals and birds of many kinds roost amongst it branches, reminding one of the poor old man of whom it was said :—

"Two owls and a hen,
Four rooks and a wren,
Did all build their nests in his beard."

What to put on it.

Truly the wonders of this plant are inexhaustible, and who can fail to be impressed with a shrub that glistens and sparkles and shines and trembles, from its leader to its lowest bough, with everything that anyone can wish for at once!—fruit, flowers, sweeties, toys, books, and jewels—here a box of bon-bons for Tiny Tim; there a neat roll containing a song; or haply Miss Tully's "White Wings" waltz, for the sweet-faced "Pro" whose little feet trip as readily on errands of mercy as ever they do when she gets a chance of dancing. Here a comfortable muffler for Mother Bunch, or a packet of tobacco for Gaffer Gray's long clay. Like the "brook," one could "go on for ever" describing the advantages of the phenonenon under discussion, and no doubt all who read are puzzling their heads to know what variety of plant this is, and where they can behold one. Every German homestead contains one at Christmas-time in full bloom, every copse-wood in Old England produces them in the raw state, and anyone in towns wishing to purchase them should immediately apply to the nearest nursery gardener, not forgetting just to mention the matter to the man at the toy-shop, the fruiterer, and the bookseller, also to call on Father Christmas, and ask him for that magic wand which shall produce one outburst of glorious bloom on Christmas Eve, when all should be thankfulness for the goodwill that reigneth upon the earth.

C. B. M.

---

*Doctor's Wife:* "Well, Barney, how is your wife?"

*Hibernian:* "Very bad, ma'am, I thank you."

*Doctor's Wife:* "Why didn't you come for the doctor?"

*Hibernian:* "Shure, I knocked, and no one came."

*Doctor's Wife:* "You should have rung the night-bell, of course."

*Hibernian:* "Oh, is it me that would be disturbin' his honour at that time of noight?"

